# The role of fathers and care-giving arrangements in informal settlements in Kenya and Ethiopia

**DOI:** 10.3389/fpubh.2023.1099568

**Published:** 2023-07-13

**Authors:** George Evans Owino, Moges Yigezu

**Affiliations:** ^1^Kenyatta University, Nairobi, Kenya; ^2^The Africa Early Childhood Network, Nairobi, Kenya; ^3^Department of Linguistics and Philology, Addis Ababa University, Addis Ababa, Ethiopia

**Keywords:** childcare, Kenya, Ethiopia, father involvement, childcare arrangements, caregiving

## Abstract

**Introduction:**

Quality childcare has been associated with multiple long-term benefits for children including improved school readiness, better educational outcomes and improved health and productivity. Evidence suggests that returns on investment are much higher when targeted at the youngest children, especially during the first 1,000 days. Despite the evidence and the ever-increasing need and potential benefits, investments made so far to make high-quality childcare accessible to the neediest families are not commensurate. It is estimated that nearly 350 million eligible pre-primary school-age children have no access to quality childcare, especially in low-and middle-income countries. The purpose of this study was to establish the role of fathers and the childcare arrangements in selected urban informal settlements in Kenya and Ethiopia.

**Methods:**

A mixed methods design was adopted with quantitative data being collected using a structured household questionnaire administered to 635 mothers of children of 0–3 years from both countries. In both countries, data was collected from three vulnerable communities namely urban informal settlements, large commercial agricultural settlements associated with flower farms, and female penal institutions where women with young children below 4 years old are incarcerated. Quantitative data was not collected from the penal institutions because during the time of the study (at the height of the Covid-19 pandemic), access to members of the public including researchers was restricted and so here only qualitative data was collected. The data reported in this article therefore does not include data from penal institutions.

**Results:**

Findings show that fathers played a major role in childcare according to 74% of respondents in Kenya and 57.7% in Ethiopia. This involvement is mainly defined in terms of providing financial support for basic needs for the family and child and for accessing health care. Some fathers were found to be either minimally involved or not involved at all. Key reasons advanced for minimal engagement included fathers having either left home permanently, had another family, was working far from home or was working long hours. Findings regarding care arrangements established that significant proportions of children had been left behind without adult supervision. Neighbors and siblings younger than 18 years provided most of the alternative care. House helps also accounted for 20.3% of care in Ethiopia with none being registered for Kenya. Daycare services only accounted for 13.4% of care in Kenya and 6.3% in Ethiopia, respectively.

**Conclusion:**

The findings revealed that fathers are fairly involved in childcare even mainly through providing necessary resources. Significantly high proportions of children were left under the care of their underage siblings leading to questions of the safety of these children. Parents and guardians in these settlements had access to a mix of care arrangements including both unpaid and paid neighbors, toddler’s siblings and in limited cases, daycare services. The low levels of utilization of daycare services indicate either limited services or inability to pay for the same. It is recommended that governments consider investing in childcare services in informal settlements.

## Introduction

Early childhood is a crucial period that requires due attention and a great deal of investment; it sets the foundation for life and has enormous impact on children’s readiness for primary school (1). Various research outputs underlined that if children are not given timely and adequate opportunities for good nutrition and adequate stimulation, they lose opportunities for good physical and intellectual growth that may not be easily compensated at any other stage in their life course. These impacts last long into adulthood and impact on the health, behaviors and learning abilities of children as adults. According to UNESCO (2), failing to provide children with better nutrition, health care and education, at this stage of development, deprives them of their right to develop as productive citizens, enjoy a better life and eventually contributes to society’s growth.

It has also been emphasized that the returns on investment in ECD are substantial (3). Hence, the combined attainments in health, nutritional, educational and social development during early childhood do not only generate benefits to individual children and families but it also saves public money through better school achievements that reduce wastage in education, greater income and taxes from skilled and economically productive individuals, healthy and responsible citizenship that reduces costs of ill health, anti-social behaviors and social inequities. ECD is, therefore, the most effective and cost-efficient time to ensure that all children develop to their full potential (4).

Samman et al. (5) assert that due to the changing relationship of women with regard to their engagement with the labor market, women in recent decades are forced to seek external support for childcare. One of these supports should naturally come from the fathers who are essential part of the child’s world. Studies reveal a specialized gender division of labour between men and women whereby men play a more instrumental role which includes providing money for basic needs, such as food and other necessities. They also support money to access health care services. Female caregivers including mothers on the other hand, play a mostly nurturing role that include stimulating, soothing, feeding, bathing and playing with the child (6–9). Father involvement has been found to encounter some barriers such as being engaged away at work, traditional gender roles that assign caregiving to females, lack of willingness on the part of fathers and lack of caregiving skills (9).

The current study tries to establish the role of fathers in childcare and the childcare arrangements available to parents of young children in selected urban informal settlements in Nairobi, Kenya and Addis Ababa, Ethiopia. For the purpose of this study, two vulnerable communities in informal settlements were selected from each country, namely, *Shiromeda* (an informal urban settlement in Addis Ababa) and *Batu/Zeway* (a rural commercial agricultural community) from Ethiopia and *Naivasha* and *Kawangware* in Nairobi, Kenya.

The study adopted a cross-sectional mixed methods research design that involves the collection of both quantitative and qualitative data. For the quantitative phase of the study, a questionnaire was developed to serve as the data-collection instrument which was used to interview the participants. The instruments developed for collecting qualitative data were structured interviews, FDG and observation guidelines. The questionnaires were administered to a total of 635 mothers or caregivers of children of 0–3 years of age from both countries.

## Materials and methods

For this study, a concurrent parallel mixed methods research design was adopted. In this design, both quantitative and qualitative data are collected and analyzed simultaneously. Thereafter, the findings are compared to determine whether they complement or contradict each other (10). A mixed methods design was preferred because through it, the quantitative and qualitative methodologies complement, mutually reinforce, and offset the biases inherent in each. More specifically, combining the two facilitated the generation of broader and deeper insights about the status of childcare in Kenya and Ethiopia (10).

The study was guided by a dual peer review process involving a research technical team and a research advisory team (RAT). The research technical team consisted of research experts and was responsible for the development of an appropriate research design, data collection, analysis, report writing and dissemination. The research advisory team comprised key members of the ECD ecosystem in each country with the mandate to advise the technical team on scientific rigour, engagement with the communities, local authorities, and national government. The RAT was updated on the progress being made in the study.

### Study site and scope

This was a dual-country study conducted in Kenya and Ethiopia and targeted mothers of children of ages 0–3 years living in vulnerable communities. Three specific types of vulnerable communities for this study were urban informal settlements, large commercial agricultural settlements associated with flower farms, and female penal institutions. These female penal institutions hold women who are accompanied with their young children. In Kenya for instance, the Prisons Act provides that if alternative care arrangements are not available, women who are sentenced to serve a prison sentence may bring their young children provided they are below 4 years of age. We purposively selected one community to represent each of these special categories. The selected sites are presented in Table 1.

**Table 1:** 

Study site	Ethiopia	Kenya
Large commercial agricultural settlements	Zeeway flower farms	Naivasha flower farms
Urban informal settlements	Shiromeda area	Dagoretti north (Kawangware)
Women’s Prison community	Kaliti central correction Centre	Langata women’s prison

### Study population and sample size

The main population for this study was mothers of children aged 0–3 years from informal settlements, large scale commercial agricultural communities and female penal institutions. Other populations interviewed included daycare centre owners, community health workers, volunteer children’s officers, prisons officials, civil society organisation personnel engaged in childcare, government officials in agencies and departments dealing with early childhood and flower farm representatives. The distribution of sample size by country and type of data collection method is presented in Table 2.

**Table 2:** 

Method of data collection	Ethiopia	Kenya	Total
Questionnaire	311	324	635
Semi-structured interviews	36	49	85
Focus group discussions	4	12	16
ITERs	0	17	17

### Methods and procedures of data collection

Data was collected through four methods and took place between February and May 2020 in Ethiopia and between August 2020 to March 2021 in Kenya. During the period of the study, institutionalized daycare centres in Kenya and Ethiopia had been closed due to the Covid-19 pandemic leading to an upsurge in homebased daycares. The entire process for carrying out the study was similar in both countries with involvement of key stakeholders including key government ministries of interior, education, health and social services, civil society organizations, faith-based leaders and other actors from each country based on the selection of three settlements. Data reported for this study is that derived from the household questionnaire which was not administered in penal institutions where only semi-structured interviews were conducted. Given that in data fathers do not have unrestricted access to their incarcerated wives and the children who accompany their mothers to prison, their role is only limited to open days when they can visit these facilities. Otherwise, the fathers have no regular contact with the children.

Quantitative data was collected using structured household questionnaires which was administered with the help of trained and experienced research assistants. The questionnaires were digitally administered in Kenya by use of the Open Data Toolkit (ODK) by use of handheld digital devices. In Ethiopia, administration was through paper-based questionnaires due to problems with access to reliable electricity and internet in the areas of the study. The qualitative data was collected through focus group discussions (FGDs) and semi-structured interviews. FGDs were held with mothers of 0–3 years, community health workers, and daycare operators. Each FGD comprised of between 6–8 participants and lasted an average of one-and-a-half hours. A total of 16 FGDs were conducted across the two countries (4 in Ethiopia and 12 in Kenya). The groups were homogeneous to avoid the disruptive nature of probable conflict of ideas that may arise if heterogeneous groups were used [homogeneity was attained by having groups comprised of one gender and of people who had similar experiences such as being parents of children 0–3 years (11)]. In this study, role of fathers was defined to mean whether they played a major, minor or no role. For cases where fathers played a minor or no role, the study proceeded to establish the reason as to why they played a minor role. Additionally, the study sought to establish the level of financial support of the father whether he catered for 100, 75, 50, 25% or 0%.

Semi-structured interviews [*n* = 85 (36 in Ethiopia and 49 in Kenya)] were conducted with key informants including government officials, prisons officers, civil society personnel and representatives of flower farms from the two countries. Other participants in the interviews included mothers of 0-3-year-old children, daycare owners, community health volunteers (CHVs), volunteer children’s officers, and local government officials. The intention of these interviews was to capture detailed information from personal experiences of mothers and from frontline workers and administrators which could not have been captured by use of the structured household questionnaires. We adopted multiple methods of data collection to generate a robust triangulated account of childcare in these areas.

Quality of services in home-based daycare centres was assessed using an adapted version of the Revised Infant Toddler Environment Rating Scale (ITERS-R). “ITERS-R is designed to assess center-based childcare programs for infants and toddlers up to 30 months of age. The Scale consists of 39 items organized into 7 subscales: space and furnishings, personal care routines, listening and talking, activities, interaction, program structure, and parents and staff” (12).

This tool was used to assess the physical environment, personal care routines, listening and talking, activities to support child development, interactions, programme structure, provisions for parents and key issues related to the staff. The rating was done for home-based daycare centres that were operating at the time of the study and in each facility, we had a team of two well-trained research assistants who did their ratings independently and were overseen by a qualified expert in conducting quality assessments. A total of 17 daycare centres were observed in Kenya. This assessment was not done in Ethiopia as these types of facilities were either lacking in the area or were closed due to COVID-19-related lockdown.

### Data processing and analysis

Quantitative data was converted to SPSS files from ODK to facilitate descriptive and inferential analysis. Basic descriptive statistics such as percentages, frequencies, and means were generated to present demographic data. The data is presented in form of graphs, charts and tables. The qualitative data, mainly collected in audio form was transcribed into text, imported to MAXQDA data analysis software, and thematically coded and analysed based on the objectives of the study. Qualitative data is presented in the form of thematic syntheses and verbatim quotations.

For the ITERs, rating of all the scores in the two counties was done during the observation by the research assistants (RAs) before leaving the daycare centres as per the recommendations from the tool. The hard copies of the responses were later typed into an excel file. The principal investigator went through the data to ensure they were complete. The ITERs assessment data was analysed descriptively (frequencies, means and medians) to describe the data from the quality assessment tool.

## Results

### The role and involvement of a father in childcare

In this regard, the study was interested in establishing the level of involvement of fathers in childcare. In this section, we shall examine the role of father in terms of whether they perform a major, minor or no role in childcare and the reasons why the father plays a minor or no role. We shall also assess the level of financial support from the fathers, and frequency of fathers’ engagement in childcare activities. Financial support of the father was seen as critical because given the nature of fathers occupations, they may sometimes not be staying together most of the time with their families but maybe far away at work. Even when they stay with the family, they may be leaving home early and arriving late. This means the other way they may contribute is in material terms and not necessarily in nurturance.

**Figure 1:**
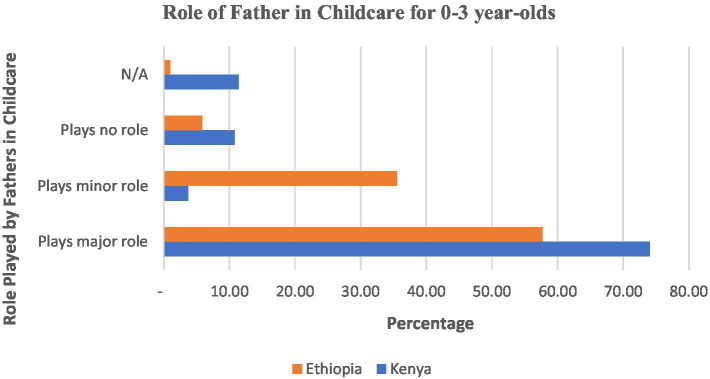
Role of father in childcare for 0–3 year-olds.

In order to examine the role and contribution of a father in child rearing, respondents were asked whether the father plays a major role, a minor role, or plays no role in childcare. With major, minor or minimal role, what is meant is the intensity of the engagement with the support to child upbringing. Major role was taken to mean that they provide the financial resources on a regular basis to their families and are dutifully performing this role. The question was posited to find out the level of involvement of a father in upbringing of a child, if not involved, the reasons for not being involved, the financial contribution of the father and the time the father spends with the child. Figure 1 summarizes the involvement of fathers in childcare.

The respondents were asked as a general perception question, whether they thought the fathers played a major, minor or no role in providing care for their children. This was intended to gauge, from a bigger perspective without going into details, what they thought the contribution of fathers to childcare was. As can be seen from Figure 1, more than half of the respondents (57%; *N* = 307 in Ethiopia and 74%; *N* = 324 in Kenya), indicated that the father plays a major role in upbringing young children aged 0–3 years while a significant number of participants (35.5% in Ethiopia) said the father plays a minor role in upbringing young children under the age of three. For those who play no role in child rearing, the greatest number (10.80%) was in Kenya against 5.86% in Ethiopia.

### Reason why father plays a minor role

**Figure 2:**
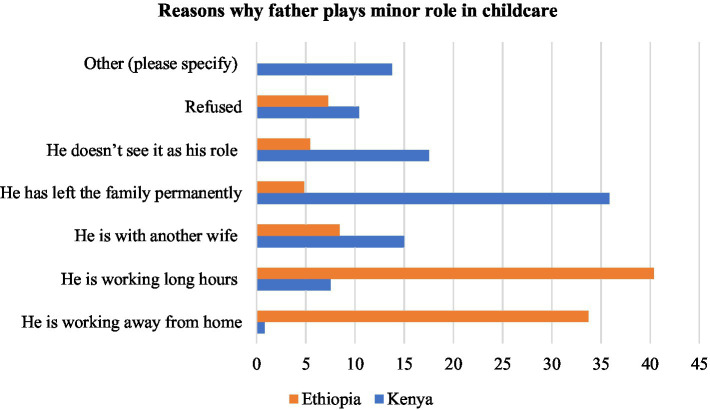
Reasons why father plays minor role in childcare.

The reasons advanced for the minor role being played or for the absence of a father in taking care of the child varied across the two countries. The central reasons mentioned by respondents from Ethiopia were that he is “working long hours” (40.36%) and that he is “working away from home” (33.73% in Ethiopia) as shown in Figure 2. In Kenya, the chief reasons are that he “has left home permanently” (35.83%) or “he does not see it as his role” (17.5%). The other reasons pointed out by the respondents include: ‘he is with another wife’, which at15.3% is more significant in Kenya compared to Ethiopia where it stands at 8.43%. Approximately 10.42% in Kenya do have refused ‘to accept as his responsibility’. These facts illustrate that fathers are absent in two ways: (a) they are absent from the homes their children live, and (b) they are absent from the health, social, and educational services designed to improve the well-being of their children (13, p. 152–153). This is illustrated in Figure 2.

Data obtained from structured interviews and focus group discussions also confirmed the reasons behind the absence of fathers from the involvement in childcare. A member of the management of one of the flower farms who was interviewed on childcare issues commented on the absence of fathers from the health and emotional wellbeing of the child as follows:

*Traditionally, the house chores are left for women. Men are mobile; they take risks and move from place to place, for various reasons. Hence, most of the domestic work as well as the responsibility of raising children rests on women. Even in cases where both parents are here [in the company], it is the mother who is more emotionally affected and become responsive when their children are ill. Even among our colleagues, who are parents, I have never seen the fathers calling home and checking on their children. It is the mothers who check if their children are well, fed properly, asleep, etc. I would be happy to see such mindset is changed and men are also involved in taking care of their children*.

Although the absence of men from the childcare and child upbringing at a close level is the most common pattern in the study areas, the entire picture is not gloomy as there are few exceptional cases that have been reported. A woman working in a flower farm and interviewed for this study happened to have understanding and responsible husband. She recounts:

*Many of them [her work-mates] do not have anyone to leave their babies with. Some would beg neighbors to babysit their children, but when the mothers return, they find their babies covered with dirt. The mothers then will take the babies home and shower and change them. As my husband babysits her, I have a peace of mind. Other kids face a wide range of challenges. Some mothers bring their sisters and brothers from countryside to babysit their babies. I myself went to the countryside and tried to find a relative to babysit my baby, but I couldn’t find one. Then, my husband decided to stop working and stay home for her*.

### Fathers’ financial contribution to childcare

**Figure 3:**
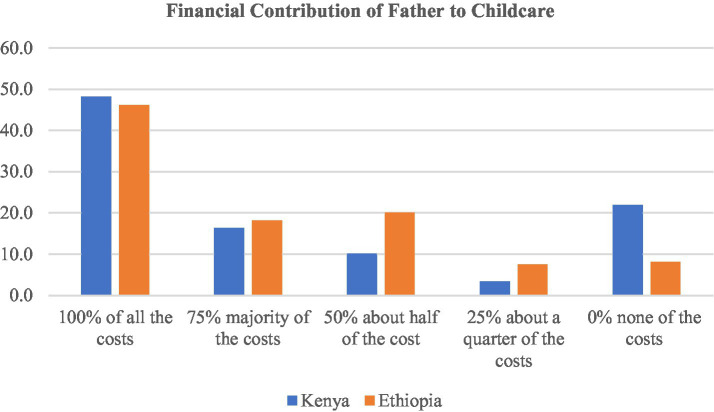
Financial contribution of father to childcare.

The involvement of men in the care of young children has direct benefits to children, one of which is resource allocation. The financial contribution of the father for child upbringing has been reported to be significant in that about half of the fathers (48.1% in Kenya and 46.10% in Ethiopia) cover all the costs of the child. Kenya, at 21.9% compared to Ethiopia at 8.2% has the highest proportion of fathers that are reported to contribute nothing financially to the upkeep of their three-year-old children. This information is illustrated in Figure 3.

### Frequency of fathers engagement in child upbringing

Fathers play a prominent role in the development of their children. As such, we sought to know how frequently fathers were involved with their children in play, in interaction with them.

**Figure 4:**
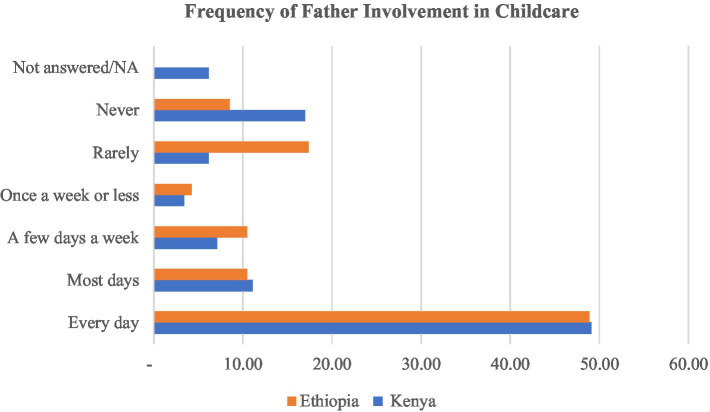
Frequency of father involvement in childcare.

According to information contained in Figure 4, Nearly equal proportions of the respondents (48.85 and 49.07% for Ethiopia and Kenya respectively) described the involvement frequency of the father in child upbringing as occurring daily. Yet a significant proportion of the caregivers stated that the father rarely or never gets involved in child upbringing. Some 8.52% of mothers from Ethiopia and 16.98% from Kenya reported that the father never engages himself with the child. These could be the fathers who are either working away from home or who have other wives.

### Caregiving arrangements

As per the interviews conducted with some mothers, there are some informal home-based daycare services which come up now and then run by individuals in the neighborhoods who baby-sit young children. in the Kenyan arm of the study, a quality assessment was conducted in 17 home-based daycare centers. These informal arrangements happened to be sub-standard as the care takers have neither the knowledge nor the facility required to take care of young children. A woman working in a flower farm and interviewed has shared the following regarding the potential risk involved with informal baby-sitters.

*I had a neighbor who had a daycare. I don’t know what she was going through, one day, she committed suicide, throwing herself from a water tank tower. When people arrived, they found a suicide note stating that she committed suicide. The kids she was looking after were on the open space, all by themselves. Anything could have happened to the children, as they had no one keeping an eye on them. You see, leaving your children even at such facilities is not free of risks*.

On the use of older siblings and relatives as babysitters, a member of the management of one of the flower farms who was interviewed has the following to say:

*Sometimes, mothers leave their babies with very young children, who themselves would normally need baby-sitters. I have recently seen such a case where the baby-sitter was hardly older than the one being watched. Children would be highly affected if they do not get adequate food. How could children as young as these ones properly serve even prepared food, let alone cooking and feeding the babies they baby-sit or themselves? This is hard to imagine… It is because there are no such facilities around this area that mothers are forced to leave their babies to very young baby-sitters, who are not older and more mature than the babies they baby sit*.

A mother who employed an 11-year-old baby-sitter for her 7-month-old child tells how she negotiated with the mother of the babysitter to keep the baby-sitter out of school.

*How do you babysit your child?*

*I have a maid, who also babysits him.*

*How old is she?*

*She is 11 years old.*

*Doesn’t she go to school?*

She is out of school at the moment.

Is she a drop out?

Yes. I begged her mother to let her stay with me; and the mother agreed.

She is just 11 years old; does she properly babysit your baby?

Yes, she can. As for feeding the baby, I am the one who prepares food and everything. All she has to do is feed the baby. I wake up 5am in the morning and get everything prepared until 7am.

A related issue to the left alone children or those who have been left with older siblings and relatives is the lack of breastfeeding policy provision that stipulates women’s right to breastfeed their children. Studies show that “labor policies in many sub-Saharan African countries remain silent on women’s right to breastfeed their children. For example, Ethiopia, Kenya, South Africa and Namibia make no legal provision for working mothers to breastfeed - even though the last has a national agenda aimed at promoting exclusive breast-feeding for the first 6 months of children’s lives” [(5), p. 49].

In commercialized flower farms considered in this study, there are no crèche provisions within the compound of the flower farms that facilitate the breastfeeding of children of the working mothers. But the managements of the flower farms have allowed mothers with young children to be off duty for an hour so that they can breastfeed their children. Feeding children in the middle of the working day appears to be inconvenient to most mothers as the 1 hour off-duty also include transport time as well feeding time.

A woman who breast-feeds her child within the permitted one-hour time describes her situation as follows:

*Is your house far away from here?*

*Yes, it is far.*

*How long does it take you to walk there?*

*Half an hour.*

*Does that mean, a two-way walk takes you an hour?*

*Yes.*

*So, how can you manage to breastfeed your baby?*

*The break time is not adequate. But, since it’s the decision of the company, what could we do? I am not able to properly breastfeed my baby or take rest for myself. The break time is very short.*

An accident which is related to breastfeeding of a child in a rush has also been reported by a work-mate.

*Very recently, a mother returned from home, having breastfed her baby. When she got here, she was called back and when she got home, she found her bay died from the breast milk he choked on. We are always in rush for work. We leave our children home and run. It is hard to pay close attention to our babies when we are in such a rush. This mother thought that her baby was asleep. The babysitter didn’t notice either. It was the baby’s uncle who found out that the baby was dead, when trying to wake him up. The mother could not even afford to pay for the essentials of the funeral. Her workmates had to contribute towards the expenses. There are lots of problems of this kind. The company does not care for our children.*

#### Network of families, neighbors, and community members

Due to the total absence of childcare services in the study area, childcare services are predominantly home-based. Other than the mother, many other people are involved in childcare services. As can be gathered from Table 3 below, 64.5% of the childcare services are provided by network of families and neighbors. In the category of caregivers, the key stakeholders are unpaid neighbors, household workers, siblings and community members in that order. For instance, 43.3% of the respondents stated that they are supported by unpaid neighbors who look after their under three-year children regularly; around 11.5% of the respondents noted that older siblings (aged between 13 to 18 years) are involved in taking care of the children. While 9.7% the respondents reported that younger siblings below the age of 12 look after their young children. Studies reported there is a significant routine involvement of children aged five and above in various care-related activities in Ethiopia and the figure has been estimated to be up to 45% [(5), p. 36].

**Table 3:** 

Care givers	Every day	3–5 times per week	1–2 days per week	Less often	Not applicable
	No (%)	No (%)	No (%)	No (%)	No (%)
Neighbors (not paid)	43 (14.0)	9 (2.9)	9 (2.9)	72 (23.5)	174 (56.7)
Neighbors (paid)	3 (1.0)	2 (0.7)	3 (1.0)	12 (3.9)	286 (93.5)
House help	58 (19.0)	3 (1.0)	1 (0.3)	10 (3.3)	234 (76.5)
Siblings aged below 12 years	22 (7.3)	2 (0.7)	0 (0.0)	5 (1.7)	274 (90.4)
Child-to-child care	0 (0.0)	1 (0.3)	1 (0.3)	5 (1.6)	299 (97.7)
Siblings aged 13-18 years	14 (4.6)	3 (1.0)	4 (1.3)	14 (4.6)	271 (88.6)
A day-care/ baby care center	6 (2.0)	1 (0.3)	3 (1.0)	5 (1.6)	291 (95.1)
An ECD center	2 (0.7)	4 (1.3)	3 (1.0)	7 (2.3)	290 (94.8)
Pre-primary school	5 (1.6)	4 (1.3)	6 (2.0)	5 (1.6)	286 (93.5)
Someone else	15 (4.9)	7 (2.3)	3 (1.0)	16 (5.2)	265 (86.6)

While much of the childcare work is done by family members and neighbors, 23.6% of the respondents reported that they depend on paid caregivers to take care of their babies. Only 4.9% claim to use childcare centers and 6.5% pre-primary schools. Given the total absence of daycare centers and pre-primary facilities that can take care of children between 0–3 years of age, it is possible that these respondents have accessed the informal childcare arrangements. Interviews conducted with female workers that moved from their home villages to the area of commercialized farms in search of jobs has shown that they brought their younger siblings to help them as caregivers to their infants, which is the case of unpaid care work that also forces the younger siblings to become dropouts from school. In the absence of older siblings to step in, it is common in Ethiopia to ‘foster’ poorer relatives in exchange for support for schooling (Ibid, p. 40).

Neighbors happened to be the main actors in filling the gap created by the total absence of childcare services in vulnerable communities. The questionnaire interview has further revealed that 43.3% of the respondents involve their neighbors (unpaid) to look after their 0–3-year-old children; 14% on daily basis, 5.8% intermittently per week and 23.45% less often. All respondents from Shiromeda site reported that they do not pay for neighbors who involve in childcare services.

In Kenya, most of the providers of alternative childcare and in whose custody, mothers would leave their children include siblings below 16 years old who accounted for 40% of the caregivers. This means these siblings have to either drop out or skip school. Other significant caregivers are unpaid neighbours (18%) and siblings of 16 years and above as is indicated in Table 4.

**Table 4:** 

Caregivers left with children	Kawangware		Naivasha		Total	
	*N*	Percent	*N*	Percent	*N*	Percent
Neighbours (not paid)	18	32.1%	1	4.8%	19	18%
Neighbours (paid)	1	1.8%	3	14.3%	4	8%
Siblings aged below 16	15	26.8%	11	52.4%	26	40%
Siblings aged 16 or above	14	25.0%	1	4.8%	15	15%
A daycare/ babycare centre	7	12.5%	3	14.3%	10	13%
Other	1	1.8%	2	9.5%	3	6%
Total	56	100.0%	21	100.0%	77	100%

The category with the lowest uptake as alternative caregivers for mothers in the study sites in Kenya are the neighbours that are paid.

## Discussion

### The father in childcare

This study which was conducted both in Ethiopia and Kenya revealed that fathers are fairly involved in child upbringing though mostly not through direct one-to-one involvement but through providing necessary resources. Only 57.7% in Ethiopia and 74% in Kenya play a major role in the provision of childcare; while around 35 and 5.86% of fathers in Ethiopia play a minor role or no role in the upbringing of their children aged 0–3 years, respectively. Fathers are absent from the provision of childcare in two ways. Either they are physically absent from the homes of their children live or they are emotionally absent from the health, social and educational services designed to improve the well-being of their children. The frequency of involvement of fathers shows that only about half of the fathers (48% in Ethiopia) involve regularly in the upbringing of their children. Such levels of the absence of fathers, physical or emotional, in the childcaring and nurturing is unfavorable to the well-being of children.

The availability and involvement of fathers is believed to contribute to the emotional well-being of children in general; and father’s presence has the benefits of reducing aggressive behavior of boys and increasing the self-esteem in girls [(13), p. 157]. Hence, how often the father spends time with the child would be beneficial to the well-being of the child.

The role of a father in upbringing young children is a vital component of child development. In Ethiopia and Kenya, as in other African countries, care responsibility is considered to be the burden of the mother. But the burden of care on women has serious implications on their earnings, their productivity, and type of work they do, their chance of being employed. This care burden also has a negative effect on mother’s participation in the workplace (5). Studies have shown that the absence or exclusion of a father from childcare is detrimental to children’s development because “men are an essential part of a child’s world; men need to hold up half of the child’s sky” [(13), p. 151].

According to African culture, both parents play different but complementary roles in childcare and child upbringing. Mothers, being the ones who bear children play mostly a nurturing role whereas fathers play an instrumental role of providing the means of sustenance as well as security (14). However, in the contemporary society, the roles have become somewhat blurred, and mothers as well, play a significant role in providing for their households.

On the other hand, due to the changing relationship of women with regard to their engagement with the labor market, the burden of care on women has posed serious implications in the life of millions of mothers and the society at large. The upshot of these challenges is that, due to the competing pressures on adult’s time and resources, significantly high proportions of children were left alone without adult supervision. Close to 40% of mothers reported that they are forced to leave their young children under three with others. Similarly, young children are left with their old siblings and relatives at an early age; and the age of older siblings and relatives a child can be left with is as high as 16 years and as low as 4 years which put the safety of the children in question.

Quite many children in many parts of the world are not receiving adequate care due to the competing pressures on adult’s time and resources. It has been reported in the literature that in 53 low- and middle-income countries 20% of children under five were without adult care and they are either left alone or in the care of a sibling under the age of ten [(5), p. 14–15]. The critical factors contributing to a decision to leave children without adult care include parental unavailability (particularly lack of involvement of fathers) and poor working conditions, limited support networks, and the inability to afford childcare services (Ibid).

Childcare services are predominantly home-based due to the nearly total absence of childcare services in informal settlements under study in Ethiopia or lack of affordable childcare services in both Kenya and Ethiopia. As a result, childcare services are mainly provided by a network of families and neighbors. Parents and guardians in the informal settlements under discussion had access to a mix of care arrangements including both unpaid and paid neighbors, young siblings, and in a limited cases daycare services.

### Recommendations

It is recommended that governments at different levels consider investing in childcare services in informal settlements. Government intervention is recommended to move childcare from a mere business enterprise towards a center for child development. The ECD policy as well as the family law and the labor market policies in Ethiopia in particular are either silent or indifferent on some pertinent issues of ECD such as extended maternity benefits (that includes the informal sector), policy on paternity and parental leaves as well as policy on crèche provisions. There is, therefore, a need to instigate serious changes in care-related policies by making labor market policies sensitive to care.

## Data availability statement

The original contributions presented in the study are included in the article/supplementary material, further inquiries can be directed to the corresponding author.

## Ethics statement

The studies involving human participants were reviewed and approved by Kenyatta University Ethical Review Committee and Institute of Ethiopian Studies of Addis Ababa University. The patients/participants provided their written informed consent to participate in this study.

## Author contributions

GO and MY were the two principal investigators for this study and contributed equally to this work including overseeing study design, data collection and analysis and synthesis and writing and revising the research manuscript. All authors contributed to the article and approved the submitted version.

## Funding

The research reported in this article was conducted with funding from Echidna Giving.

## Conflict of interest

The authors declare that the research was conducted in the absence of any commercial or financial relationships that could be construed as a potential conflict of interest.

## Publisher’s note

All claims expressed in this article are solely those of the authors and do not necessarily represent those of their affiliated organizations, or those of the publisher, the editors and the reviewers. Any product that may be evaluated in this article, or claim that may be made by its manufacturer, is not guaranteed or endorsed by the publisher.
